# Genetic Architecture and Functional Implications of the CSF-Contacting Nucleus

**DOI:** 10.1007/s12264-023-01084-z

**Published:** 2023-07-05

**Authors:** Siyuan Song, Yumin Yuan, Lingling Xu, Jun Jiang, Ying Li, Yao Yan, Qing Li, Fang Zhou, Junli Cao, Licai Zhang

**Affiliations:** 1 Jiangsu Province Key Laboratory of Anesthesiology, Xuzhou Medical University, Xuzhou, 221004 China; 2 Jiangsu Province Key Laboratory of Anesthesia and Analgesia Application Technology, Xuzhou Medical University, Xuzhou, 221004 China; 3 NMPA Key Laboratory for Research and Evaluation of Narcotic and Psychotropic Drugs, Xuzhou, 221008 China; 4 School of Nursing, Xuzhou Medical University, Xuzhou, 221004 China

**Keywords:** Gene architecture, Cerebrospinal fluid-contacting nucleus (CSF-contacting nucleus), Biological function

## Abstract

We previously identified a unique nucleus, the cerebrospinal fluid (CSF)-contacting nucleus. This study aims to understand its gene architecture and preliminarily suggest its functions. The results showed that there were about 19,666 genes in this nucleus, of which 913 were distinct from the dorsal raphe nucleus (non-CSF contacting). The top 40 highly-expressed genes are mainly related to energy metabolism, protein synthesis, transport, secretion, and hydrolysis. The main neurotransmitter is 5-HT. The receptors of 5-HT and GABA are abundant. The channels for Cl^–^, Na^+^, K^+^, and Ca^2+^ are routinely expressed. The signaling molecules associated with the CaMK, JAK, and MAPK pathways were identified accurately. In particular, the channels of transient receptor potential associated with nociceptors and the solute carrier superfamily members associated with cell membrane transport were significantly expressed. The relationship between the main genes of the nucleus and life activities is preliminarily verified.

## Introduction

It is well-known that many physiological activities and diseases are closely related to the regulation of cerebrospinal fluid (CSF) [1, 2]. The assessment of CSF is important in the diagnosis of diseases [3, 4]. For example, changes in N-glycomics and N-glycoproteomics in the CSF provide relevant information about Alzheimer's disease [5]. The amount of free light-chain kappa in the CSF can distinguish myelitis from non-inflammatory myelitis [6]. Conversely, drug therapy and even cell transplantation through the CSF pathway may produce central effects; for example, lumbar anesthesia through the low CSF pathway may lead to central complications in the brain [7]. Physiologically, the close exchange between CSF and brain interstitial fluid, as well as the cerebrospinal fluid-brain barrier (CBB), contributes to the maintenance of central nervous system homeostasis [8]. This paradoxical phenomenon of both barriers and communication has always lacked a reasonable explanation. Both the somas and processes of the central nuclei are confined to the brain parenchyma, while the terminals of the peripheral nerves (including 31 pairs of spinal nerves and 12 pairs of cranial nerves) are only connected to the peripheral organs (e.g., muscles, bones, and blood vessels). Which nerve manages the CSF? Is there a unique neural structure that can break through the CBB and mediate the transport of informational substances from the brain to the CSF or *vice versa*? A clear explanation has been lacking. However, after 30 years of research, we have a clear answer: there is a special neural structure in the brain parenchyma that breaks through the CBB to implement two-way communication between the brain and CSF. First, we found a special tracer and method to identify the structure of the brain [9, 10]. Based on this method, we found a pair of left-right symmetrical clusters of neurons in the brain stem, with their somas located in the parenchyma and their processes extending into the CSF across the CBB. Using computer reconstruction techniques, we describe its position, adjacency, and spatial image and provide empirical formulas for predicting the coordinates of the central points of this cluster based on the body weight of the SD rat. Based on the naming conventions of nuclei in the brain, we named it the cerebrospinal fluid-contacting nucleus (CSF-contacting nucleus) [11]. This nucleus not only exists in lower animals but has also been verified in the brain of a primate, the rhesus monkey [12]. In addition, we have successfully established a model animal with CSF-contacting nucleus knockout [13] and studied the distribution of some neuroactive substances in the nucleus and their relationship with certain life activities [14–20]. However, the CSF-contacting nucleus is a new discovery and is different from known nuclei. The somas have extensive projective connections with functional nuclei in the brain [21, 22]. Their terminals extending into the CSF have sensory, release, and secretory functions [23]. Many life activities and diseases are related to the CSF. Therefore, there is still much work to be done to truly understand the biological characteristics of the CSF-contacting nucleus.

Genes are the source of life activity and expression [24]. Although gene construction alone cannot fully reveal the nature of the CSF-contacting nucleus, it is a good method to predict its physiological function according to the gene profile. Therefore, we applied high-throughput sequencing technology to reveal the genetic composition of the CSF-contacting nucleus. In addition, molecular biological techniques combined with quantitative behavioral monitoring were used to verify the correlation between the main genes, such as 5-HT, GABA, adrenomedullin, and Na^+^ ions, in the CSF-contacting nucleus and specific life activities.

## Materials and Methods

### Experimental Animals

Specific pathogen-free (SPF)-grade adult male Sprague‒Dawley rats weighing 250 ± 50 g were provided by the Experimental Animal Centre of Xuzhou Medical University, license number SCXK (Jiangsu) 2015-0009. All animal procedures were performed in accordance with the Guide for the Care and Use of Laboratory Animals and were approved by the Committee for the Ethical Use of Laboratory Animals, Xuzhou Medical University (Ethics Approval No. L20211001001). Animals were housed in a controlled environment with temperatures of 23 °C to 26 °C, light/dark cycles of 12 h each, and free access to food and water.

### High-Throughput Sequencing and Analysis

The rats were anesthetized by intraperitoneal injection of pentobarbital sodium (40 mg/kg), normal saline was perfused through the left heart, and the brain was collected after decapitation. The brain segment containing the CSF-contacting nucleus and the dorsal raphe nucleus (DRN) was placed in cold artificial CSF and cut on a vibratome at 300 μm. Under a stereomicroscope, the CSF-contacting nucleus and DRN were extracted from coronal brain slices by 12-needle microdissection. Samples rich in these two nuclei were treated as follows: mRNA was extracted using TRIzol reagent (Invitrogen, USA); RNA concentration and mass were then measured using the Agilent 2100 Bioanalyzer (Agilent Technologies, USA), NanoDrop (Thermo Fisher, USA), and 1% agarose electrophoresis; a library was constructed with 1 µg of total RNA (RNA integrity number) ≥ 8.

High-throughput sequencing of the constructed library was performed using Illumina HiSeq (Illumina, USA) (GENEWIZ, Suzhou, China). The normalized fragments per kilobase of transcript per million mapped reads (FPKM) value was used to represent the expression level of each gene. Gene Ontology (GO) and Kyoto Encyclopedia of Genes and Genomes (KEGG) were used for gene enrichment analysis of cell functions and signaling pathways [25, 26].

Differential gene expression analysis of the CSF-contacting nucleus and DRN was also applied. The unique reads with an absolute fold change value ≥ 1.5 and a P value < 0.05 were used as the thresholds to define the differentially-expressed genes (DEGs) in the pairwise comparisons.

### Immunofluorescence (IF)

After anesthesia by the intraperitoneal injection of sodium pentobarbital (40 mg/kg), the rats were perfused with 4% paraformaldehyde through the left ventricle. The brain segment where the nucleus was located was placed in 4% paraformaldehyde and fixed for 12 h. The tissue was then dehydrated in 30% sucrose until it sank. Coronal frozen sections were cut through the CSF-contacting nucleus at a thickness of 40 μm on a freezing microtome. The sections were placed in 0.1 mol/L PBS for CB-594 IF double-label staining. According to different experimental purposes, different primary antibodies were used first. These included rabbit anti-microtubule associated protein 2 (MAP2) (Abcam, USA, Cat# ab32454), rabbit anti-synapsin (Abcam, USA, Cat# ab64581), rabbit anti-TPH2 (Proteintech, China, Cat# 22590-1-AP), goat anti-5-HT (Abcam, USA, Cat# ab66047), rabbit anti-GABA_B_ (Santa Cruz, USA, Cat# sc-14006), rabbit anti-ADM (Affinity, China, Cat# DF8501), and rabbit anti-c-Fos (Cell Signaling Technology, USA, Cat# 2250). To assess the damage to the CSF-contacting nucleus caused by Cholera toxin subunit B with Saponin (CB-SAP), a goat anti-CB primary antibody (List Labs, USA, Cat# 703) was added. The sections were incubated while protected from light at 4 °C overnight, after which Alexa Fluor 488 secondary antibodies diluted 1:200 (Life Technologies, USA, Cat# A-21206 or Cat# A-11055 according to the species of primary antibody) were added and incubated with the sections at room temperature for 2 h. After IF staining, the sections were mounted on slides and the cover slipped using an antifade mounting solution. A confocal laser scanning microscope (LSM 880, Zeiss, Germany) was used to photograph the positively-stained structures.

### Western Blotting (WB)

Rats were anesthetized by intraperitoneal injection of sodium pentobarbital (40 mg/kg), and then the brain was extracted rapidly at low temperature after decapitation. The CSF-contacting nucleus was separated. RIPA lysis buffer (500 μL/200 mg tissue) with PMSF (1 mmol/L) was added to digest the brain tissue. The supernatant of digested tissue was homogenized by ultrasound on ice, centrifuged at 4 °C at 12,000 r/min for 15 min, and the supernatant was stored at − 80 °C. The protein concentration of the extracted protein was determined by a BCA protein assay kit and then adjusted to the same concentration within the different groups. Equivalent protein samples were separated by 12% sodium dodecyl sulfate-polyacrylamide gel electrophoresis and transferred to a PVDF membrane. The transferred PVDF membrane was blocked with 5% skimmed milk at room temperature for 2 h. Primary anti-GABA B (1:800, Santa Cruz, USA, Cat# sc-14006), anti-ADM (1:500, Affinity, China, Cat# DF8501), and anti-β-actin antibodies (1:1000, Proteintech, China, Cat# 66009-1-Ig) were added to the PVDF membrane and incubated at 4 °C overnight. After warming to room temperature for 30 min, the cell membranes were rinsed with washing buffer 3 times for 5 min each, and then an HRP-conjugated secondary antibody (1:1000, Cell Signaling Technology, USA, Cat# 7074 or Cat# 7076 according to the species of primary antibody) was added and incubated for 2 h at room temperature. After rinsing with washing buffer 3 times for 5 min each, the membrane was developed by ECL substrate (Thermo Scientific, 32106) and exposed by the ChemiDoc XRS system with Image Lab software (Bio-Rad). Then, the results were analyzed by ImageJ software.

### Establishment of the Inflammatory Pain Model and Behavioral Testing

One hundred microliters of complete Freund’s adjuvant (CFA) was subcutaneously injected into the left hind paw of the rats to prepare the inflammatory pain model. The thermal withdrawal latency was applied according to the Hargreaves method [27]. Rats were exposed to a heat radiation stimulator (IITC Life Science Inc., Woodland Hills, CA). The time from the beginning of heat radiation to the emergence of leg-raising avoidance was the thermal withdrawal latency. The automatic cutoff time was set at 30 s to prevent tissue damage.

### Establishment of the Chronic Stress Model and Behavioral Testing

The rats were restrained for 1 h every day for 1 week in a homemade plastic bottle. After the end of daily restraint, the rats were put back into the cage. The effects of chronic immobilization stress (CIS) were measured by an open-field test. The open-field experiment used a black box 40 cm high and 100 cm long. The bottom of the open field box was divided into nine small squares. Immediately above was the camera; the field of view covered the entire open field. The rats were placed in the central grid, and the activity state of rats in a 5 min period was analyzed by a computer tracing analysis system. The laboratory was quiet, at a temperature of ~ 20 °C and with sufficient light. The stationary times, distances moved, and the number of crossings were counted.

### Sodium Sensing and Sodium Appetite Testing

First, 1 μL of 0.1% CB-594 was injected into the lateral ventricle. After 48 h, 10 μL 0.5 mol/L NaCl was also injected into the lateral ventricle. One hour later, the rats were perfused, and CB/c-Fos double labeling in the CSF-contacting nucleus was detected by IF. For sodium appetite testing, both spontaneous and induced sodium intakes were measured. (1) Spontaneous sodium intake: rats were supplied with 0.3 mol/L NaCl solution and distilled water every day. The volume of 0.3 mol/L NaCl solution drunk was recorded and converted into mL/100 g. (2) Induced sodium intake: rats were subcutaneously injected with 10 mg/kg furosemide and 5 mg/kg captopril (FURO + CAP) without food and water for 60 min and then supplied with 0.3 mol/L NaCl solution and distilled water. The volume of 0.3 mol/L NaCl drunk was measured as described above. For both spontaneous and induced sodium intake, the salt preference value was calculated by the formula NaCl (vol)/NaCl (vol) + distilled water (vol) × 100 %.

### Statistics

SPSS 16.0 software was used for statistical analysis. The data are expressed as the mean ± SD or mean ± SE. Student's *t*-test or the Mann‒Whitney test was used for between-group comparisons, as appropriate. One-way ANOVA was used for comparisons among different groups, followed by the Student-Newman‒Keuls *post hoc* test. Two-way ANOVA with repeated measurements was used to test the behavioral results at different time points followed by Tukey’s *post hoc* test. GraphPad Prism 7.0 software was used to generate graphs. A *P* value < 0.05 was considered significant. DESeq2 was used for differential expression analysis. GOseq (v1.34.1) was used to identify GO terms that annotate a list of enriched genes. KEGG pathways were identified by using R software.

## Results

### The Genetic Architecture of the CSF-contacting Nucleus

#### Total Genes in the CSF-contacting Nucleus

According to the standardized FPKM values, the total number of genes in the CSF-contacting nucleus was ~ 19,666. Among these genes, 1337 (6.8%) had FPKM values > 60; 4886 (24.84%) had FPKM values between 15 and 60; and 5997 (30.49%) had FPKM values between 3 and 15. A total of 2519 genes (12.81%) had FPKM values between 1 and 3. A total of 3820 genes (19.42%) had FPKM values between 0.1 and 1; 1107 genes (5.63%) had FPKM values between 0 and 0.1. For details, see Table 1.

**Table 1:** Total genes in the CSF-contacting nucleus

Sample	FPKM 0–0.1	FPKM 0.1–1	FPKM 1–3	FPKM 3–15	FPKM 15–60	FPKM >60	Total
R1	1224 (6.18%)	3852 (19.43%)	2514 (12.68%)	6039 (30.47%)	4867 (24.55%)	1325 (6.68%)	19821
R2	1030 (5.27%)	3753 (19.20%)	2527 (12.93%)	5984 (30.62%)	4903 (25.09%)	1347 (6.89%)	19544
R3	1068 (5.44%)	3855 (19.63%)	2516 (12.81%)	5967 (30.39%)	4888 (24.90%)	1340 (6.82%)	19634
Average	1107 (5.63%)	3820 (19.42%)	2519 (12.81%)	5997 (30.49%)	4886 (24.84%)	1337 (6.80%)	19666

#### The Top 40 Highly-Expressed Genes in the CSF-Contacting Nucleus

Based on the FPKM values, the top 40 most enriched genes in the CSF-contacting nucleus are shown in Table 2.

**Table 2:** The top 40 highly-expressed genes in the CSF-contacting nucleus

Gene Symbol	Gene ID	FPKM1	FPKM2	FPKM3	Description
Mt-co3	ENSRNOG00000030700	13507.47	12377.97	13645.94	Mitochondrially encoded cytochrome c oxidase III
Mt-co1	ENSRNOG00000034234	12904.39	11732.29	12429.73	Mitochondrially encoded cytochrome c oxidase 1
Mt-co2	ENSRNOG00000030371	12241.1	11215.5	11523.39	Mitochondrially encoded cytochrome c oxidase II
Mt-atp6	ENSRNOG00000031979	9771.39	9253.13	8755.51	Mitochondrially encoded ATP synthase 6
Mt-cyb	ENSRNOG00000031766	6904.84	6487.44	6808.65	Mitochondrially encoded cytochrome b
Mt-nd2	ENSRNOG00000031033	4807.98	4662.68	4596.67	Mitochondrially encoded NADH dehydrogenase 2
Mt-nd1	ENSRNOG00000030644	4404.45	4110.98	4163.11	Mitochondrially encoded NADH dehydrogenase 1
Cst3	ENSRNOG00000005195	2730.03	2427.77	2396.49	Cystatin C
Apoe	ENSRNOG00000018454	2337.17	2291.63	2252.18	Apolipoprotein E
Mt-nd5	ENSRNOG00000029971	2043.53	2040.43	2016.59	Mitochondrially encoded NADH dehydrogenase 5
Zwint	ENSRNOG00000048682	1841.5	1613.92	1688.92	ZW10-interacting kinetochore protein
Mt-atp8	ENSRNOG00000033299	1753.83	1793.17	1793.34	Mitochondrially encoded ATP synthase 8
S100b	ENSRNOG00000001295	1682.46	1682.61	1745.4	S100 calcium-binding protein B
Sparc	ENSRNOG00000012840	1464.68	1150.53	1227.61	Secreted protein acidic and cysteine-rich
Mir770	ENSRNOG00000040384	1449.26	1332.03	1305.98	MicroRNA 770
Nnat	ENSRNOG00000024923	1335.57	1601.31	1664.79	Neurontin
Sparcl1	ENSRNOG00000015093	1308.84	1290.66	1213.07	SPARC like 1
Nap1l5	ENSRNOG00000007808	1307.08	1298.88	1180.07	Nucleosome assembly protein 1-like 5
Mt-nd3	ENSRNOG00000033615	1272.73	1137.2	1021.66	Mitochondrially encoded NADH dehydrogenase 3
Mt-nd4l	ENSRNOG00000031053	1217.07	1066.24	984.18	Mitochondrially encoded NADH 4 L dehydrogenase
Mt-nd6	ENSRNOG00000029042	1199.63	1258.19	1113.83	Mitochondrially encoded NADH dehydrogenase 6
Tph2	ENSRNOG00000003880	1186.45	1146.27	1255.64	Tryptophan hydroxylase 2
Uchl1	ENSRNOG00000002343	1165.45	979.1	1088.29	Ubiquitin C-terminal hydrolase L1
Ndrg2	ENSRNOG00000010389	1111.05	1024.66	1018.74	NDRG family member 2
Actb	ENSRNOG00000034254	953.65	959.48	972.43	Beta-actin
Ckb	ENSRNOG00000010872	941.06	867.29	874.33	Creatine kinase B
Scg2	ENSRNOG00000015055	869.07	722.82	738.95	Secretogranin II
Hsp90ab1	ENSRNOG00000019834	827.02	753.47	750.96	Heat shock protein 90 alpha family class B member 1
Calm2	ENSRNOG00000030871	782.81	767.56	821.92	Calmodulin 2
Tmsb4x	ENSRNOG00000047931	782.01	720.91	690.9	Thymosin beta 4, X-linked
Bcat1	ENSRNOG00000015514	760.53	704.01	725.25	Branched chain amino acid transaminase 1
Aldoa	ENSRNOG00000052802	754.18	683.39	720.51	Aldolase, fructose-bisphosphate A
Calm1	ENSRNOG00000004060	717.94	782.58	709.8	Calmodulin 1
Scd2	ENSRNOG00000046005	693.04	876.49	759.51	Stearoyl-coenzyme A desaturase 2
Eef1a1	ENSRNOG00000009439	687.29	648.75	682.18	Eukaryotic translation elongation factor 1 alpha 1
Mir5125	ENSRNOG00000049077	680.44	695.78	695.34	MicroRNA 5125
Ubb	ENSRNOG00000042271	679.21	638.19	632.19	Ubiquitin B
Aldoc	ENSRNOG00000011452	664.77	496.9	599.83	Aldolase, fructose-bisphosphate C
Gm25994	ENSRNOG00000045884	653.91	665.08	618.01	Predicted gene 25994
Atp6v0c	ENSRNOG00000006542	622.59	572.8	609.29	ATPase H+-transporting V0 subunit C

#### Genes Related to Neurotransmitters, Receptors, Channels, Transporters, and Signaling Molecules in the CSF-contacting Nucleus

**Fig. 1:**
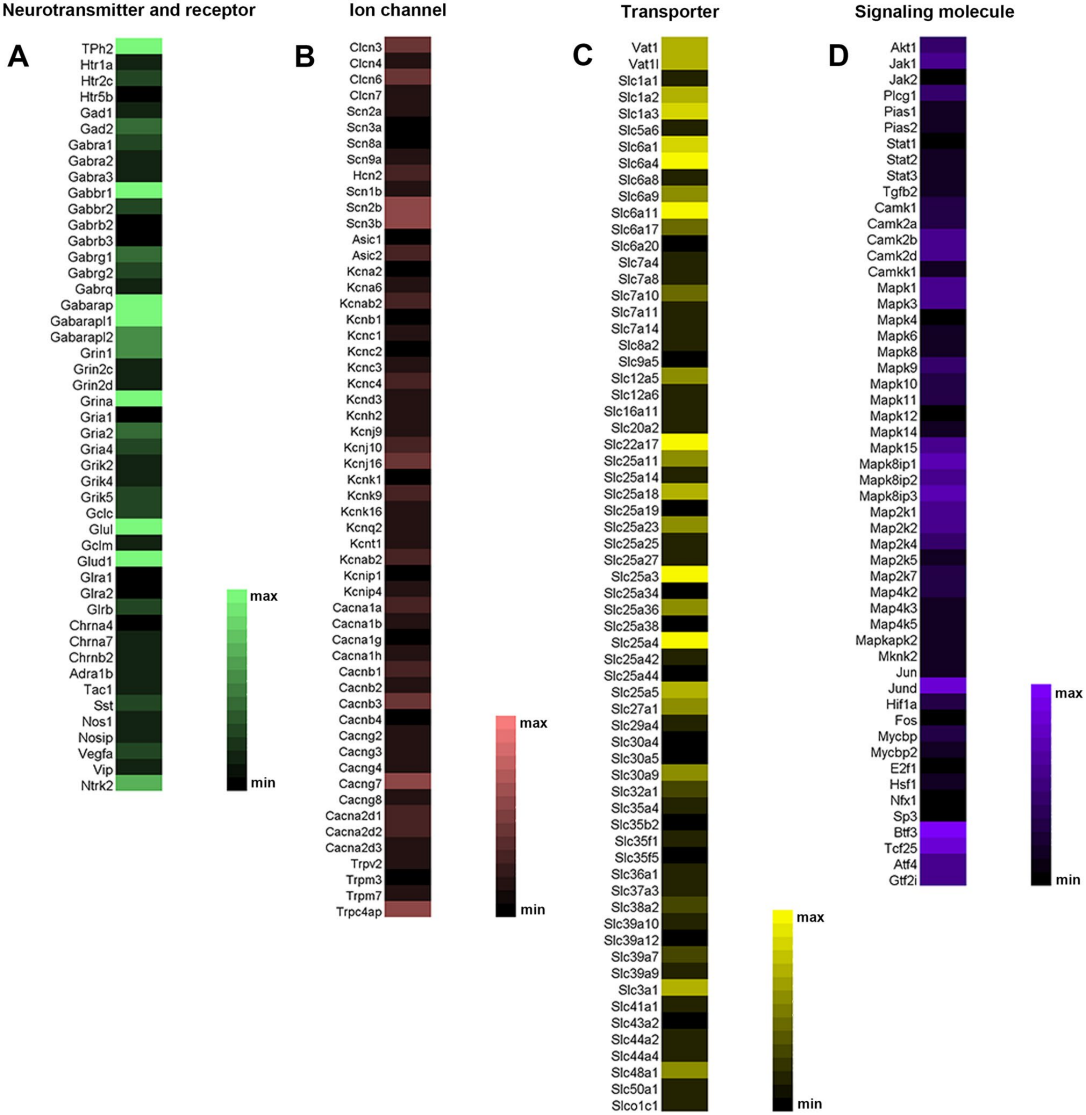
Distribution and contents of specific bioactive gene substances in the CSF-contacting nucleus. **A** Neurotransmitters and receptors. **B** Ion channels. **C** Transporters. **D** Signaling molecules. The min-max color bars represent fragments per kilobase of transcript per million mapped reads (FPKM) values.

Based on the FPKM values, 5-HT and GABA were the most abundant transmitters and receptors in the CSF-contacting nucleus. The ion channels included common Cl^−^, Na^+^, K^+^, Ca^2+^, and transient receptor potential. The transporters were mainly solute carrier superfamily members, and the signaling pathway-related molecular genes were mainly AKT, CAMK, MAPK, JUND, and BTF (Fig. 1).

#### Special Functional Analysis of Genes in the CSF-Contacting Nucleus

Three ranges of FPKM were evaluated: FPKM values > 60, FPKM values between 15 and 60, and FPKM values between 3 and 15.

GO gene enrichment was used to analyze gene ontology, including molecular functions, cell components, and biological processes.

**Fig. 2:**
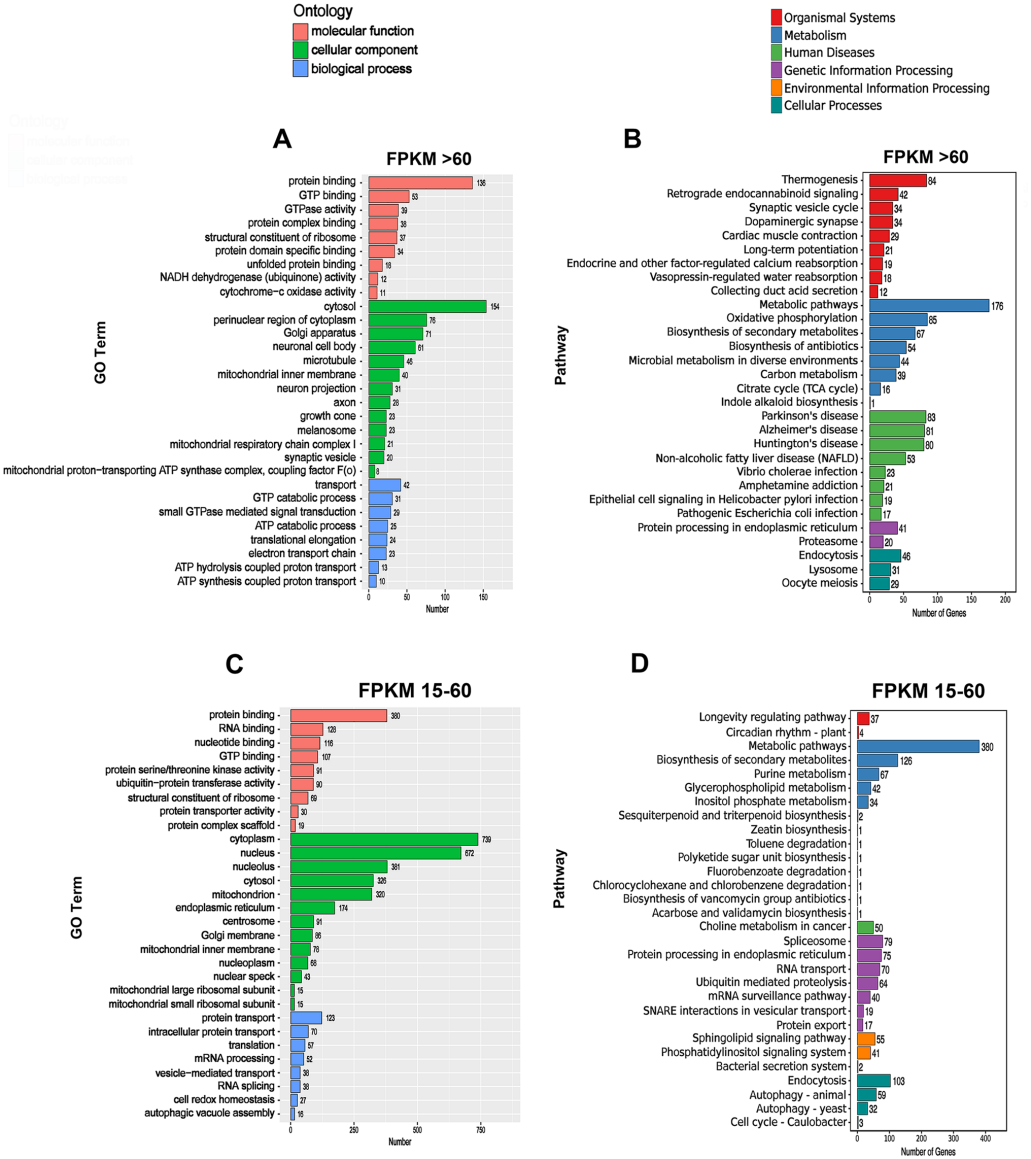

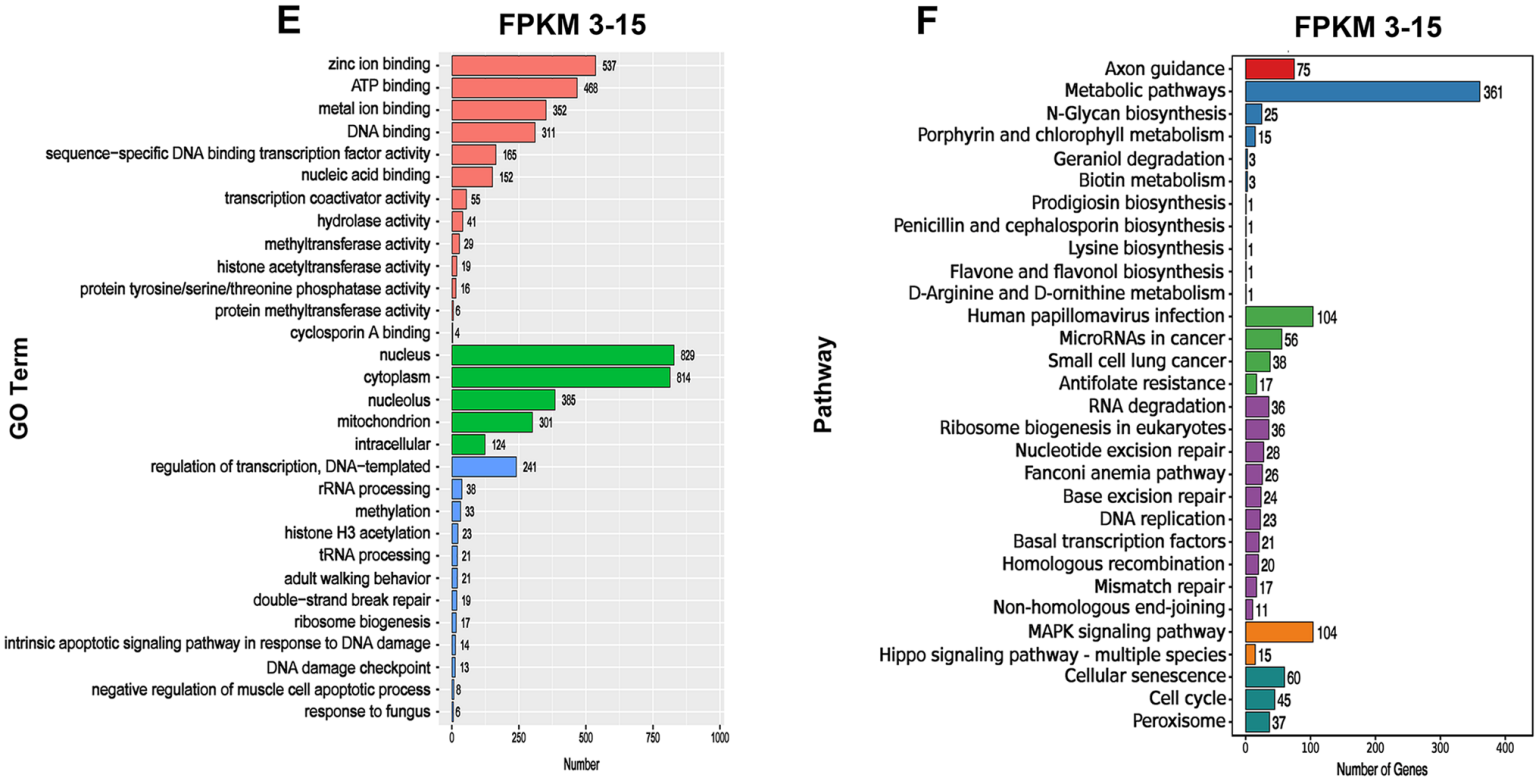
Genetic analysis of the ontology and signaling pathways in the CSF-contacting nucleus. **A, C, E** Gene Ontology (GO) analysis in the CSF-contacting nucleus at different FPKM values. Red represents the biological process genes, green represents the cellular component genes, and blue represents the biological function genes. **B, D, F** KEGG analysis in the CSF-contacting nucleus at different FPKM values. Red represents the genes of the organismal system's pathway, blue represents the genes of the metabolic pathway, green represents the genes of the personal disease pathway, purple represents the genes of the information editing pathway, orange represents the genes of the environmental information processing pathway, and cyan represents the genes of the cellular process pathway.

When the FPKM value was > 60, the highest expression of cell functional genes was protein binding, cell component genes were cell solutes, and biological process genes were transporters (Fig. 2A). When the FPKM value was between 15 and 60, the highest expression of cellular functional genes was also binding proteins, followed by RNA binding, nucleotide binding, and GTP-binding genes. The cytoplasmic component genes were mainly related to the cytoplasm, followed by nuclear, nucleolar, and mitochondrial genes. Biological process genes are mainly involved in intracellular protein transport, translation, and mRNA processing (Fig. 2C).

When the FPKM value was between 3 and 15, the functional genes with high expression were mainly involved in zinc ion binding, followed by ATP binding, metal ion binding, and DNA binding. The genes related to cell components were the nucleus, cytoplasm, nucleolus, and mitochondria. Biological process genes were mainly associated with transcriptional regulation and DNA templating, followed by rRNA processing, methylation, and histone H3 acetylation (Fig. 2E).

The gene expression of the organismal system, metabolism, human disease, genetic information processing, environmental information processing, and cellular processing in the CSF-contacting nucleus was analyzed using KEGG.

When the FPKM value was > 60, the highly-expressed genes were mainly associated with thermogenesis, metabolic pathways, Parkinson’s disease, Alzheimer's disease, Huntington's disease, non-alcoholic fatty liver, endoplasmic reticulum protein processing, and endocytosis signaling pathways (Fig. 2B).

When the FPKM value was between 15 and 60, the highly-expressed genes were mainly related to the longevity regulation pathway, metabolic pathways, choline metabolism in cancer, and signaling pathways, such as the spliceosome sphingolipid signaling system and the endocytosis pathway (Fig. 2D).

When FPKM values ranged from 3 to 15, highly-expressed genes were only associated with axon guidance, metabolic pathways, human papillomavirus infection, RNA degradation, the MAPK signaling pathway, cellular senescence, and other signaling pathways (Fig. 2f).

#### Comparison of Genes Between the CSF-contacting Nucleus and the Dorsal Raphe Nucleus

We used a |fold change| value ≥ 1.5 and a *P* value < 0.05 to select and compare the highly differentially expressed genes (DEGs) between the CSF-contacting nucleus and DRN.

**Fig. 3:**
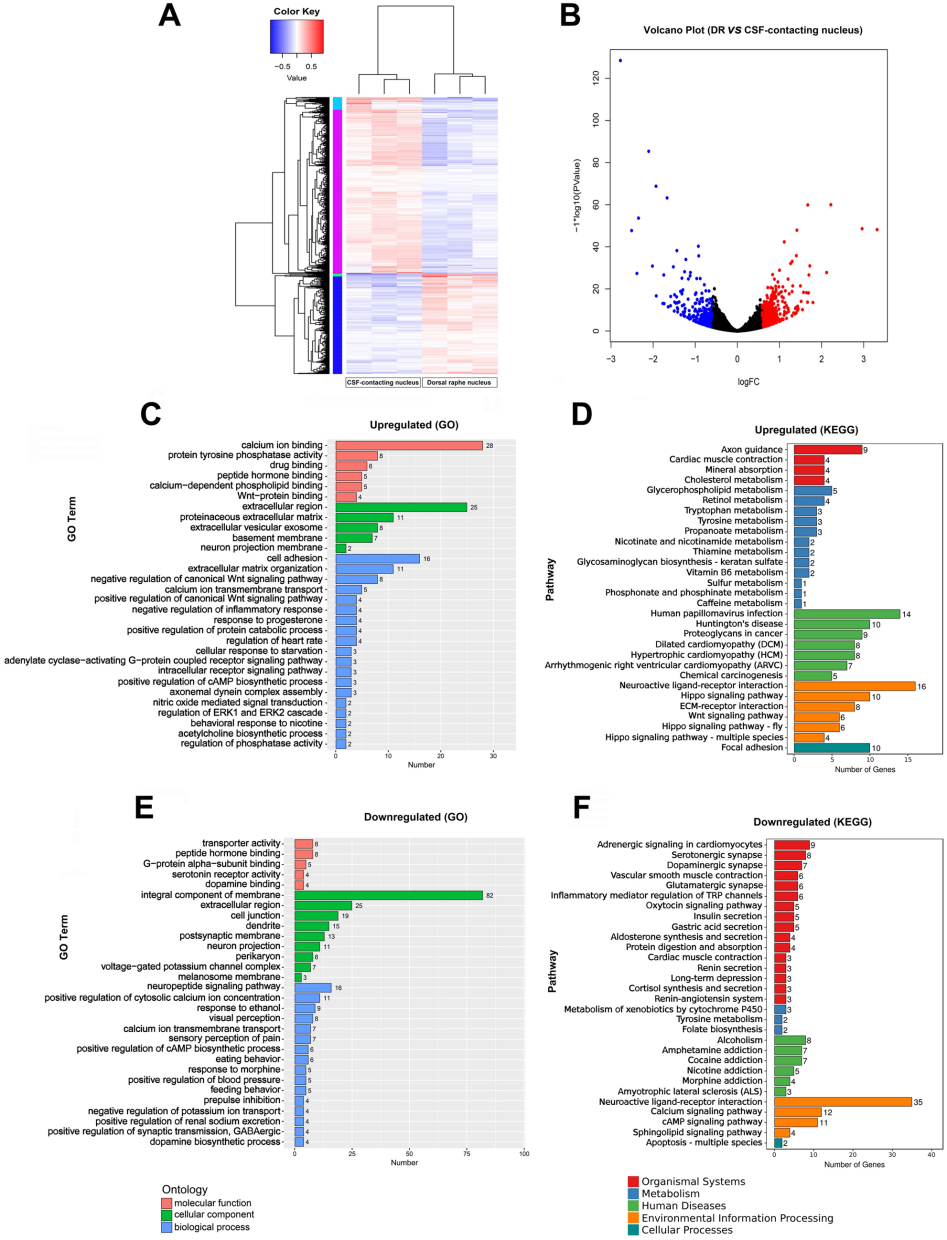
Comparison of the gene between the CSF-contacting nucleus and the dorsal raphe nucleus. **A** A heatmap of the genes differentially expressed between the CSF-contacting nucleus and the dorsal raphe nucleus. Red bars represent upregulated genes, while blue bars represent downregulated genes. **B** A volcano plot of the differentially-expressed genes. Red dots represent upregulated genes, blue dots represent downregulated genes, and black dots represent genes with no significant change in expression. **C, D** Gene Ontology (GO) and Kyoto Encyclopedia of Genes and Genomes (KEGG) functional analyses of the upregulated genes in the CSF-contacting nucleus compared with the dorsal raphe nucleus. **E, F** GO and KEGG functional analyses of the downregulated genes in the CSF-contacting nucleus compared with the dorsal raphe nucleus. The functional annotations of the genes shown in different colors are the same as those shown in Fig. 1.

Compared with the DRN, there were 913 DEGs, among which 607 were upregulated and 306 were downregulated (Fig. 3A, B). Then, we used GO analysis and KEGG pathway analysis to assess these significantly differentially expressed genes.

GO enrichment analysis of upregulated genes showed that their main molecular functions were calcium ion binding, protein tyrosine phosphorylation, and drug binding. The main components of cells were extracellular regions, protein extracellular matrix, and extracellular sac-like exosomes. The main biological functions were related to cell adhesion, extracellular matrix organization, and negative regulation of typical Wnt signaling pathways (Fig. 3C).

KEGG pathway analysis of upregulated genes revealed significant differences between the two nuclei related to axon guidance, glycerophospholipid metabolism, neuroactive ligand-receptor interactions, and focal adhesion pathways (Fig. 3D). Enrichment analysis of downregulated GO genes showed that the main molecular functions were transporter activation, peptide hormone binding, and G-protein α-subunit binding. The main components of cells were the whole membrane, extracellular region, and cell junction components. The main biological functions were related to neuropeptide signaling pathways, positive regulation of plasma calcium ion concentration, and ethanol reaction (Fig. 3E). KEGG pathway analysis of downregulated genes mainly showed association with 5-HT-ergic synapses, tyrosine metabolism, alcoholism, and neuroactive ligand-receptor interactions (Fig. 3F).

### Relation Between the Main Genes of the CSF-contacting Nucleus and Life Activities

#### 5-HT in the CSF-contacting Nucleus and Inflammatory Pain

1. Reconfirmation of the neurotransmitter 5-HT in the CSF-contacting Nucleus

**Fig. 4:**
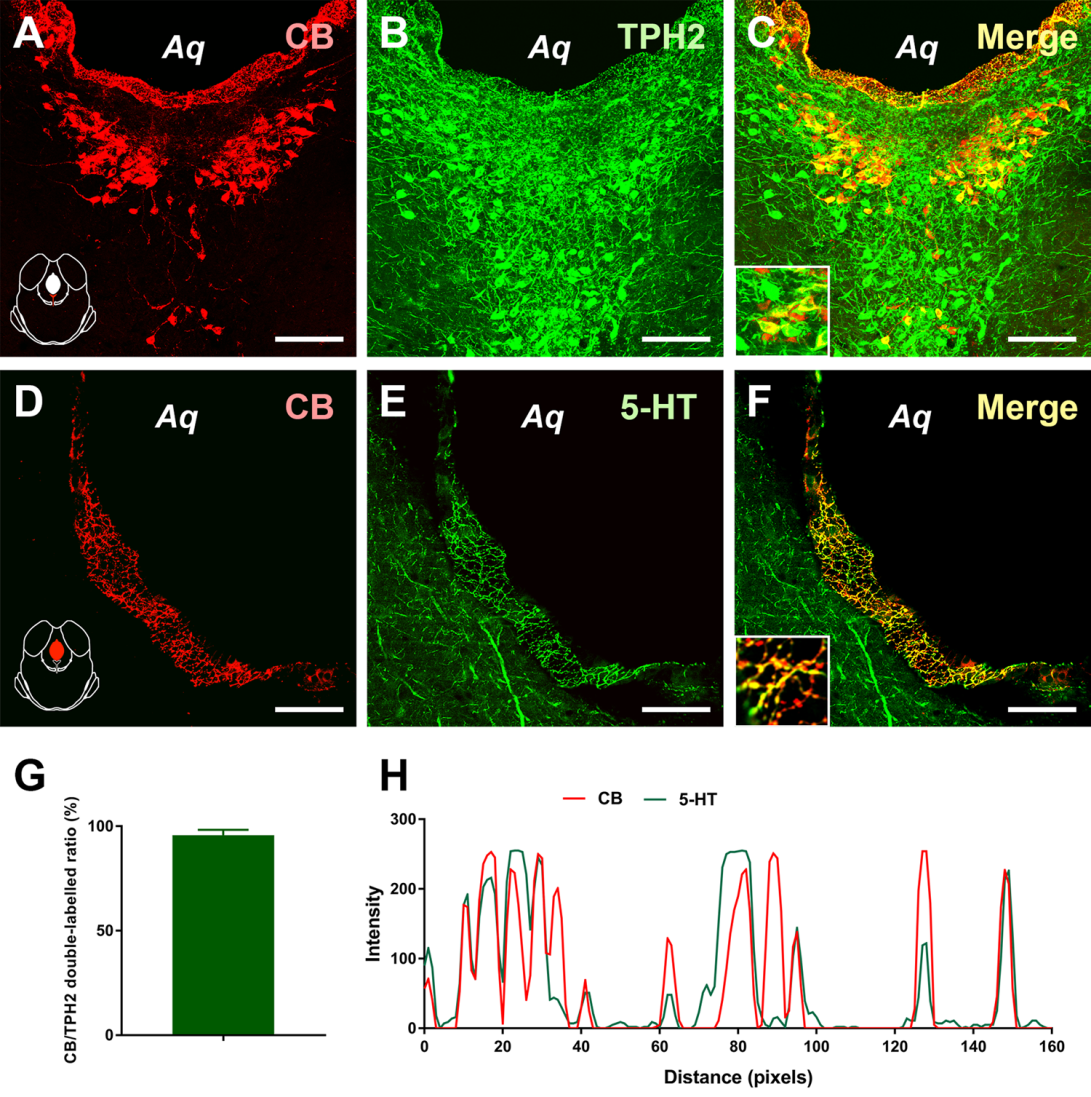
Reconfirmation and assessment of TPH_2_ and 5-HT in the CSF-contacting nucleus. **A–C** The CSF-contacting nucleus: CB-positive neurons (red), TPH_2_-positive neurons (green), and double-labeled neurons (yellow). **D**–**F** CB-positive neural axons and terminals (red), 5-HT-positive axons and terminals (green), and double-labeled axons and terminals (yellow). **G** Statistics of the ratio of CB and TPH_2_ double labeling. **H** The intensity of the CB (red line) terminals and 5-HT (green line) terminals along the ventricular wall. Aq: aqueduct. Scale bars, 100 μm (**A–C**); 25 μm (**D–F**).

As tryptophan hydroxylase 2 (TPH_2_), a synthetase of serotonin, was abundant in the CSF-contacting nucleus, we further verified this by immunofluorescence (Fig. 4). TPH_2_ accounted for 96 ± 2.66% of CB-positive neurons in the CSF-contacting nucleus. In addition, on the walls of the aqueduct, the density of 5-HT terminals was almost the same as that of the CSF-contacting nucleus.

- (2) The Relationship Between 5-HT in the CSF-contacting Nucleus and Inflammatory Pain

**Fig. 5:**
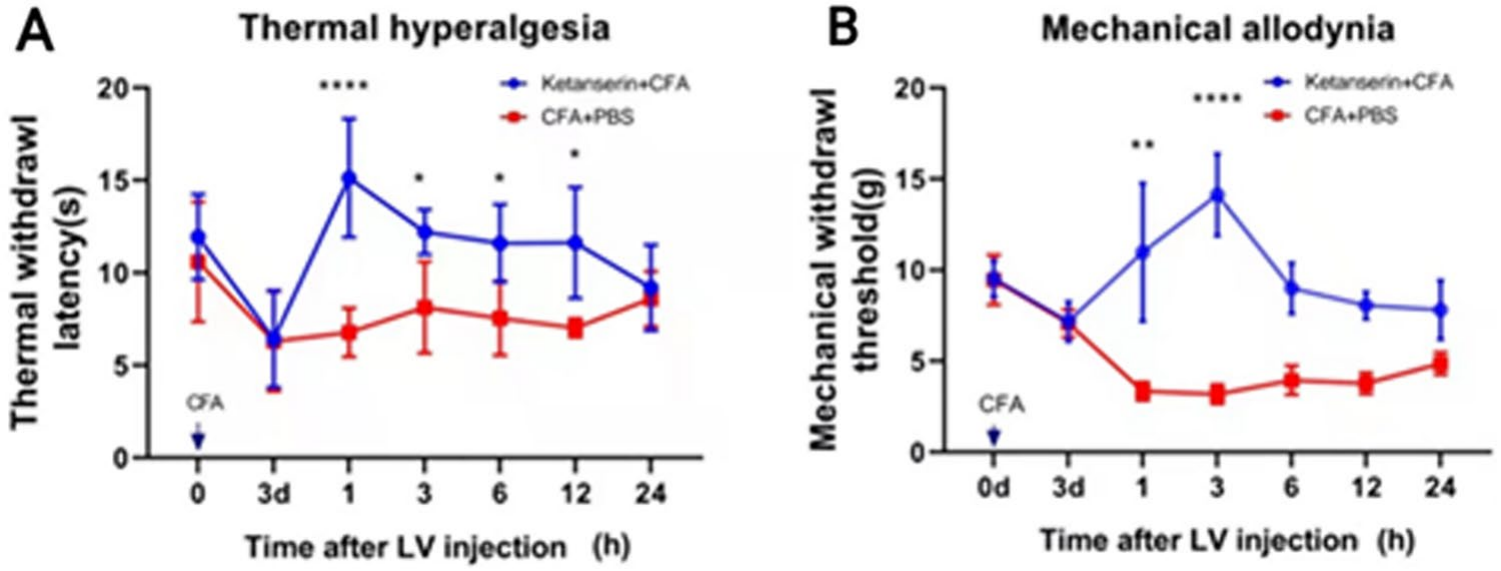
Effect of an antagonist to the 5-HT_2A_ receptor in the CSF-contacting nucleus on inflammatory pain behavior in rats. **A** The thermal withdrawal latency decreases significantly after injection of CFA into hind paws. Microinjection of the 5-HT_2A_ receptor antagonist ketanserin into the CSF-contacting nucleus significantly relieves the hyperalgesia caused by CFA (mean ± SEM, *n* = 6 rats per group, *P < 0.05, ****P < 0.0001). **B** The mechanical withdrawal latency decreases significantly after injection of CFA into hind paws. Microinjection of the 5-HT_2A_ receptor antagonist ketanserin into the CSF-contacting nucleus significantly relieves the hyperalgesia caused by CFA (mean ± SEM, *n* = 6 rats per group, **P < 0.01, ****P < 0.0001).

On days 1, 3, and 7 after hind paw administration of CFA, the paw withdrawal latency was significantly decreased compared with that on day 0. On day 3 after CFA, ketanserin, an antagonist of the 5-HT2A receptor, was injected into the lateral ventricle to antagonize the 5-HT2A receptors in the CSF-contacting nucleus of rats. At 1, 3, 6, 12, and 24 h after injection, the thermal withdrawal latency was significantly increased (two-way ANOVA, Fig. 5A). In addition, the mechanical withdrawal threshold was significantly increased in all rats (two-way ANOVA, Fig. 5B).

#### GABA_B_ Receptors in the CSF-Contacting Nucleus and Inflammatory Pain

1. GABA_B_ Receptors Reconfirmed in the CSF-contacting Nucleus with IF

**Fig. 6:**
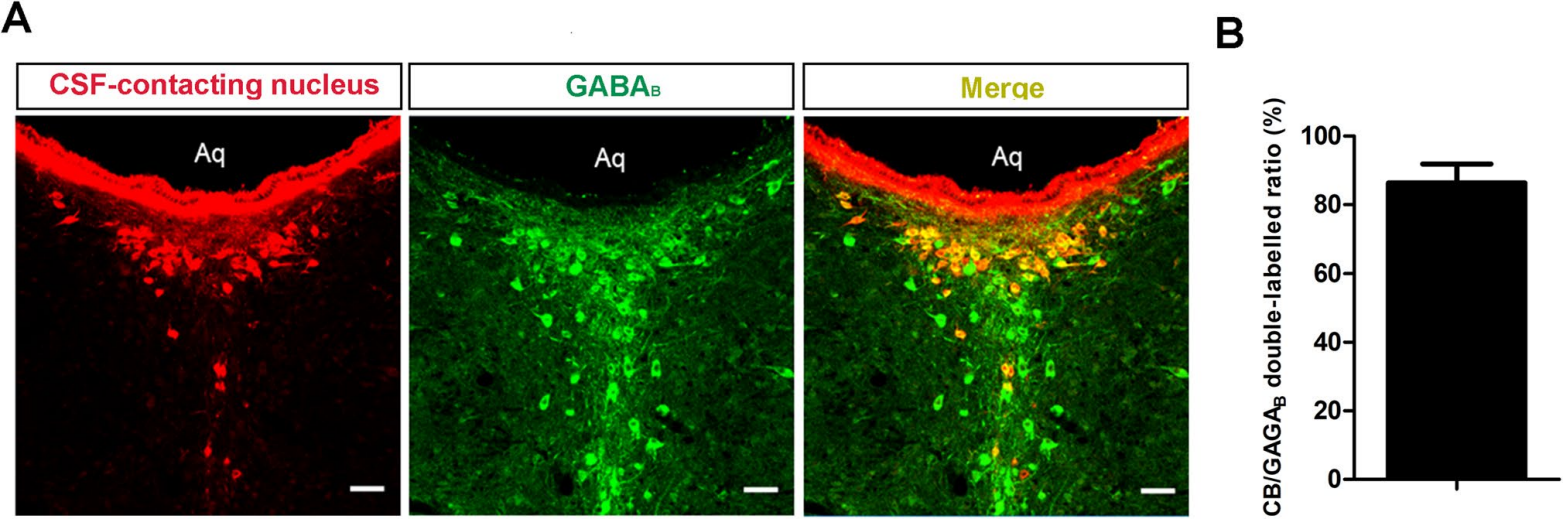
GABA_B_ receptors reconfirmed in the CSF-contacting nucleus. **A** Double labeling (yellow) of the CSF-contacting nucleus (red) and GABA_B_ receptor (green). **B** The percentage of neurons in the CSF-contacting nucleus expressing GABA_B_ receptors (mean ± SD, *n* = 6). Scale bars, 100 μm.

Based on transcriptome screening, IF showed that 86.33 ± 5.57% of neurons expressed GABA_B_ receptors (Fig. 6A, B) in the CSF-contacting nucleus.

(2) Effects of A GABA_B_ Receptor Antagonist on Inflammatory Pain Behavior

**Fig. 7:**
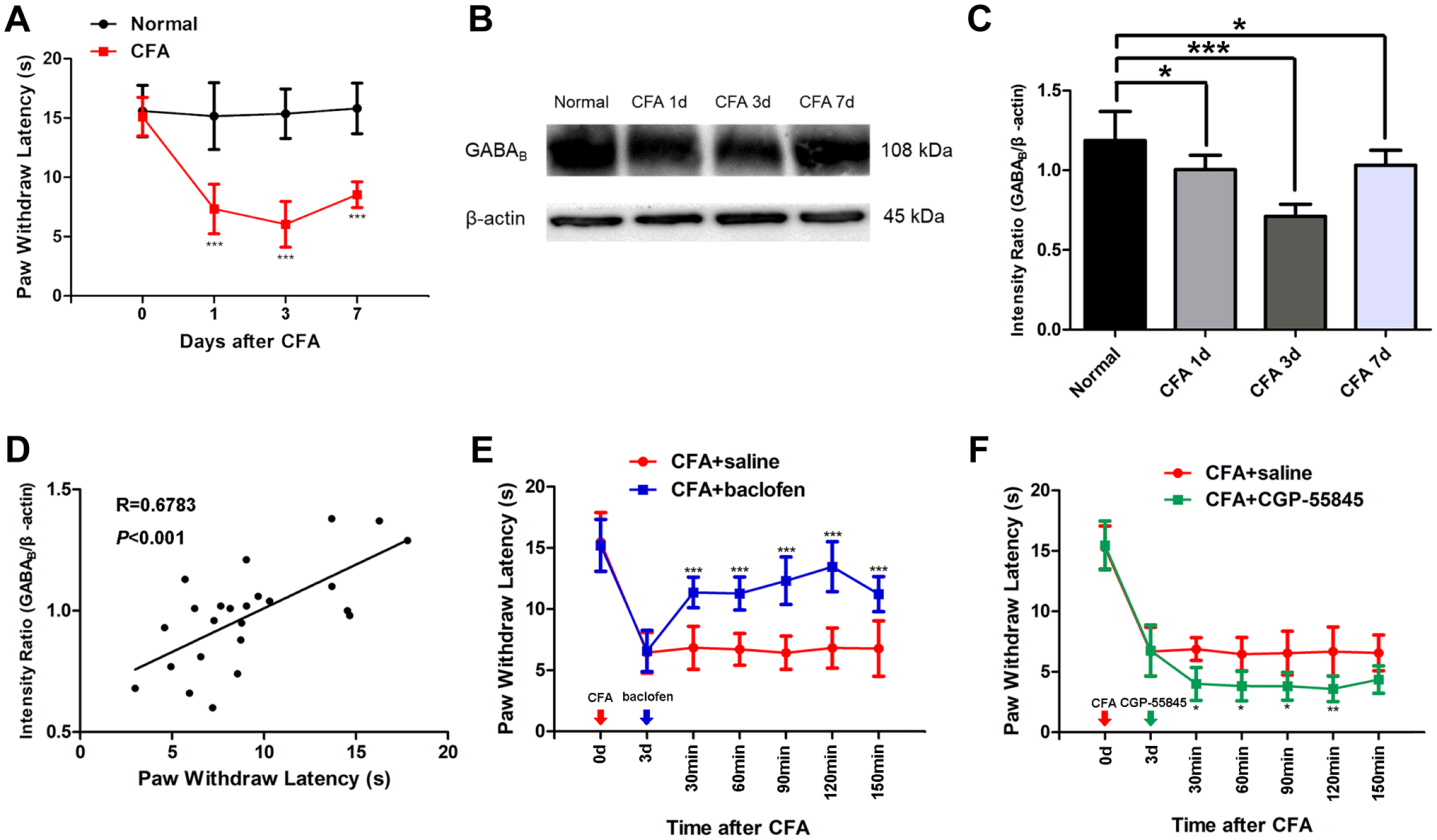
Effects of an antagonist of the GABA_B_ receptor in the CSF-contacting nucleus on inflammatory pain behavior. **A** The paw withdrawal latency decreases significantly after injection of CFA into the hind paw. (mean ± SD, *n* = 6 rats per group, ***P < 0.001). **B, C** Western blot analysis of GABA_B_ receptor expression in the CSF-contacting nucleus at different time points after CFA (mean ± SD, *n* = 6, *P < 0.05, ***P < 0.001). **D** Correlation analysis of GABA_B_ receptor expression in the CSF-contacting nucleus and the paw withdrawal latency after the injection of CFA. **E** Microinjection of the GABA_B_ receptor agonist baclofen into the CSF-contacting nucleus significantly relieves the hyperalgesia caused by CFA (mean ± SD, *n* = 6 rats per group, ***P < 0.001). **F** The GABA_B_ receptor antagonist CGP-55845 aggravates the hyperalgesia caused by CFA (mean ± SD, *n* = 6 rats per group, *P < 0.05, **P < 0.01).

On days 1, 3, and 7 after hind paw administration of CFA, the withdrawal latency was significantly decreased compared with the control group (two-way ANOVA: group × time interaction (*F*_3, 30_ = 9.487, *P* < 0.001); main effect group (*F*_1, 10_ = 224.1, *P* < 0.001), Fig. 7A). Corresponding to the results of inflammatory pain behavior, the Western blot results showed that GABA_B_ receptor expression decreased significantly at 1, 3 and 7 days after inflammatory pain (one-way ANOVA: *F*_3, 20_ = 16.91, *P* < 0.001; Fig. 7B, C) and was positively correlated with pain behavior (*r* = 0.6783, *P* < 0.001; Fig. 7D). On day 3 after CFA, baclofen, an agonist of the GABA_B_ receptor, was injected into the CSF-contacting nucleus of rats. The pain threshold was significantly increased at 30, 60, 90, 120, and 150 min after baclofen injection (two-way ANOVA: group × time interaction (*F*_6, 60_ = 7.751, *P* < 0.001); main effect group (*F*_1, 10_ = 59.18, *P* < 0.001), Fig. 7E). In contrast, CGP-55845, a GABA_B_ receptor antagonist, significantly decreased the pain threshold at 30, 60, 90 and 120 min after injection (two-way ANOVA: group × time interaction (*F*_6, 60_ = 2.446, *P* < 0.05); main effect group (*F*_1, 10_ = 26.14, *P* < 0.001), Fig. 7F).

#### Adrenomedullin in the CSF-Contacting Nucleus and Chronic Stress

**Fig. 8:**
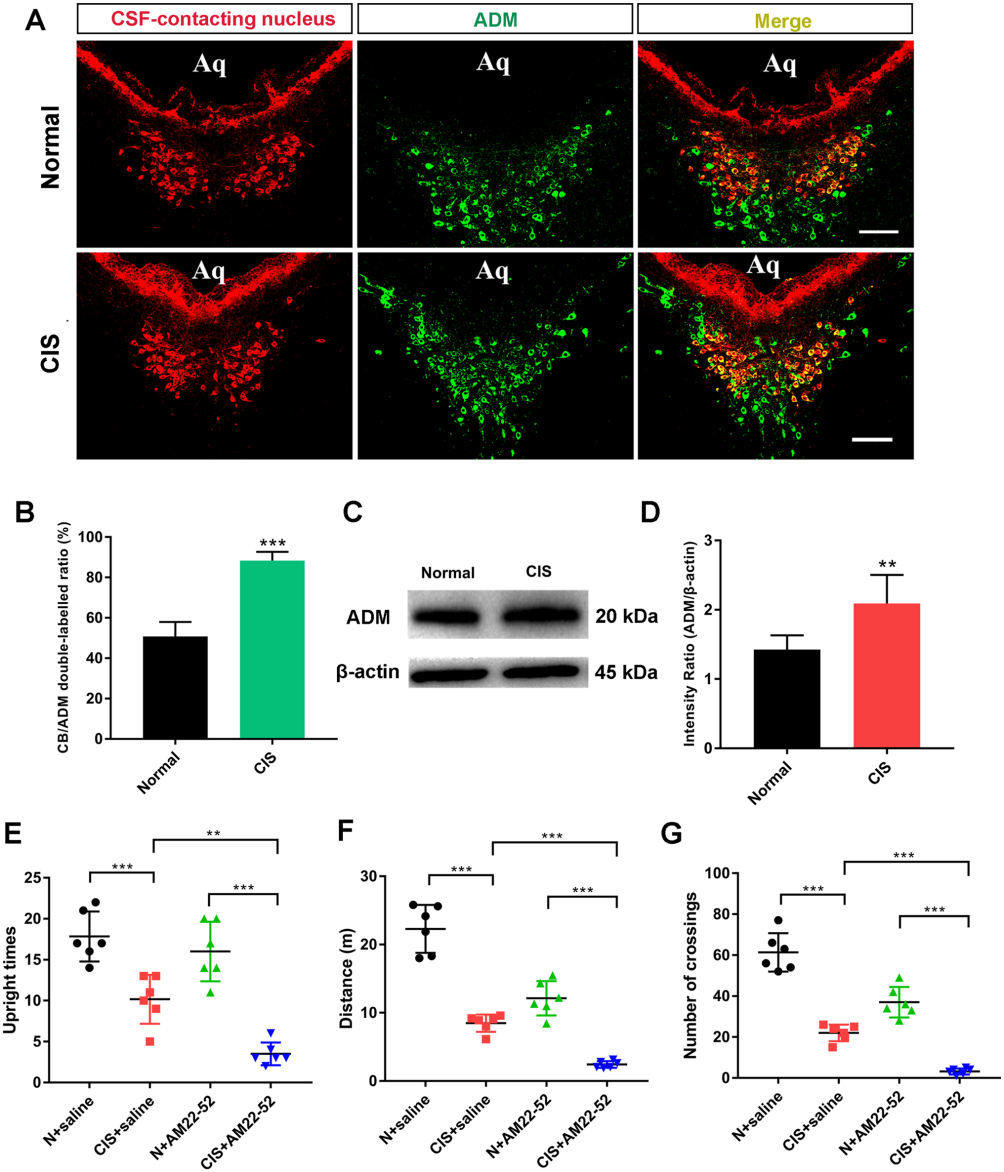
ADM in the CSF-contacting nucleus mediates chronic stress. **A** Co-labeling of the CSF-contacting nucleus (red) and ADM (green) in the normal and CIS groups. **B** Statistics of CB/ADM co-labeling changes after CIS (mean ± SD, *n* = 6 rats per group, ***P < 0.001). **C, D** Western blot analysis of ADM expression in the CSF-contacting nucleus after CIS. (mean ± SD, *n* = 6, **P < 0.01). **E, F, G** Comparison of the open field test behaviors in the N+saline, CIS+saline, N+AM22-52, and CIS+AM22-52 groups (mean ± SD, *n* = 6 rats per group, **P < 0.01, ***P < 0.001). ADM: adrenomedullin, N: normal, CIS: chronic immobilization stress. Scale bars, 100 μm.

Adrenomedullin (ADM), a protein associated with stress, was expressed in the CSF-contacting nucleus with immunofluorescence (IF) (Fig. 8A). After chronic stress, the effects on chronic stress behavior of the ADM antagonist AM22-52 and the double-labeling rate of ADM and CSF-contacting neurons increased significantly (*t*_10_ = 11.07, *P* < 0.001; Fig. 8B). The WB results were consistent with the above results. The expression of ADM was significantly increased (*t*_10_ = 3.543, *P* < 0.01; Fig. 8C, D). In the stress model, compared with the N+saline group, the stationary time, distance moved, and the number of crossings of rats in the CIS+saline group were significantly decreased (*P* < 0.001; Fig. 8E–G). After injection of AM22-52 (CIS+AM22-52 group), the stationary time (one-way ANOVA: *F*_3, 20_ = 30.05, *P* < 0.001; Fig. 8E), distance moved (one-way ANOVA: *F*_3, 20_ = 81.62, *P* < 0.001; Fig. 8F), and the number of crossings (one-way ANOVA: *F*_3, 20_ = 89.67, *P* < 0.001; Fig. 8G) were further significantly decreased after administration of the ADM antagonist AM22-52 to CIS rats.

#### The CSF-Contacting Nucleus Is Involved in Sodium Sensing and Sodium Appetite

**Fig. 9:**
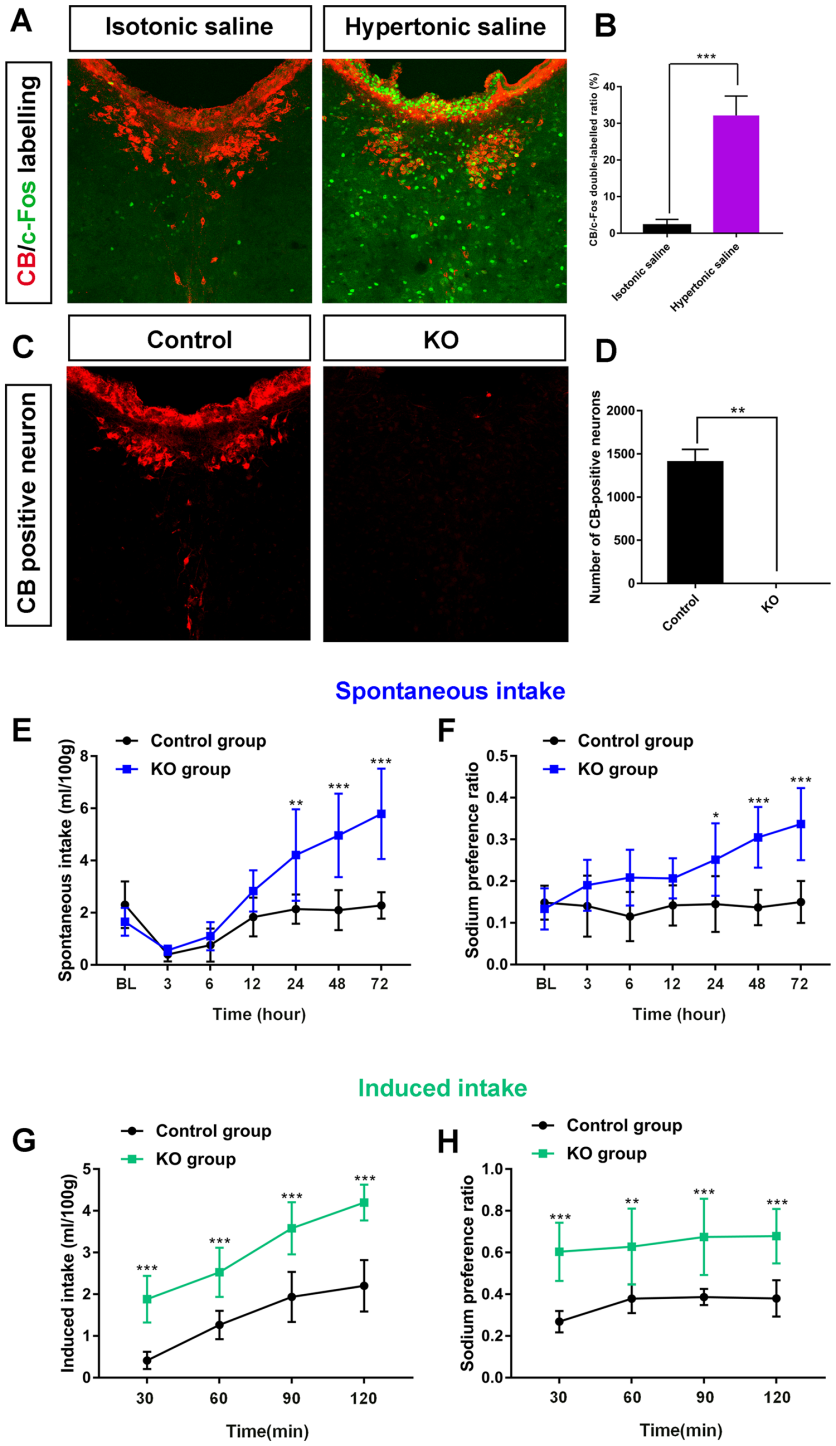
The CSF-contacting nucleus is involved in sodium sensing and sodium appetite. **A** Image of CB and c-Fos co-labeling after isotonic saline or hypertonic saline injection into the cerebrospinal fluid. **B** Statistics of CB/c-Fos co-labeling in the isotonic saline and hypertonic saline groups (mean ± SD, *n* = 6 rats per group, ***P < .001). **C** Image of CB-positive neurons in the control and CSF-contacting nucleus knockout (KO) groups. **D** Statistics of CB-positive neurons in the control and KO groups (mean ± SD, *n* = 6, **P < 0.01). **E, F** The spontaneous sodium intake (**E**) and preference ratio (**F**) in the control and KO groups (mean ± SD, *n* = 6, *P < 0.05, **P < 0.01, ***P < 0.001). **G, H** The induced sodium intake (**G**) and preference ratio (**H**) in the control and KO groups (mean ± SD, *n* = 6, **P < 0.01, ***P < 0.001). Scale bars, 100 μm.

Compared with the injection of isotonic saline into the CSF, the expression of c-Fos protein in the CSF-contacting nucleus was significantly increased after the CSF administration of hypertonic saline (*t*_10_ = 13.34, *P* < 0.001; Fig. 9A, B).

Seven days after CB-SAP was injected into the CSF, the neurons in the CSF-contacting nucleus were destroyed (Mann‒Whitney test, *P* < 0.01), thus successfully establishing an animal model of CSF-contacting nucleus knockout (KO) [13]. At 24, 36, and 72 h, the spontaneous 0.3 mol/L NaCl intake and sodium preference increased significantly in the KO group compared with the control group (two-way ANOVA: for the spontaneous intake test, group × time interaction (*F*_6, 60_ = 8.034, *P* < 0.001); main effect group (*F*_1, 10_ = 31.5, *P* < 0.001); for the sodium preference ratio test, group × time interaction (*F*_6, 60_ = 3.497, *P* < 0.01); main effect group (*F*_1, 10_ = 70.87, *P* < 0.001) (Fig. 9E, F). After subcutaneous injection of furosemide combined with low-dose captopril, the rats with CSF-contacting nucleus KO showed an enhanced induced sodium appetite. At 30, 60, 90, and 120 min, 0.3 mol/L NaCl intake, and sodium preference increased significantly in the KO group compared with the control group (two-way ANOVA: for the spontaneous intake test, group × time interaction (*F*_3, 30_ = 0.9919, *P* > 0.05); main effect group (*F*_1, 10_ = 160.6, *P* < 0.001); for the sodium preference ratio test, group × time interaction (*F*_3, 30_ = 0.2302, *P* > 0.05); main effect group (*F*_1, 10_ = 84.66, *P* < 0.001) (Fig. 9G, H).

## Discussion

**Fig. 10:**
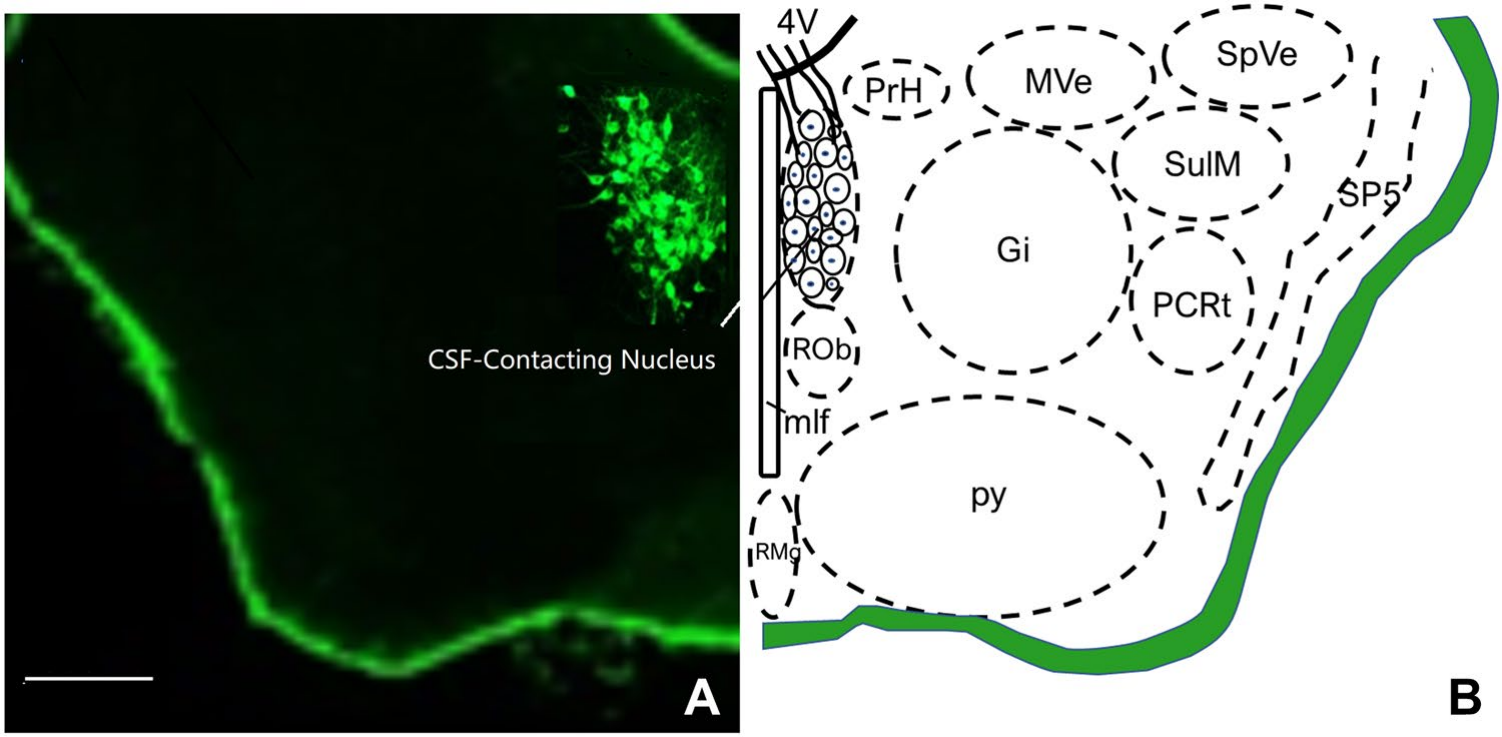
The anatomical location of the CSF-contacting nucleus. **A** Actual plane image. **B** Schematic diagram. Scale bar, 500 μm.

The somas of the CSF-contacting nucleus were located in the brain stem, adjacent to the DRN and some cranial nerve nuclei (Fig. 10), which receive projections from many nuclei in the brain [21, 22, 28, 29]. It is unique in the nervous system by having processes extended into the CSF. We even suggest that it be included as the 13th cranial nerve, which is specialized for functional regulation between the brain and CSF. Due to a wide range of physiological activities or diseases involving its regulation, a comprehensive understanding of the biological characteristics of the nucleus is of great scientific importance for both life science and medicine.

Genes, also known as genetic factors, store all the information on cell development, growth, modification, apoptosis, signaling pathways, and other processes. All life phenomena are related to genes and are also internal factors that determine health. Our study showed that ~ 19,666 genes were expressed in the CSF-contacting nucleus. This genetic information should help us understand its biological functions. However, the data set is very large, so it is difficult for us to fully reveal them given the space limitation. It is possible to select a subset of genes and suggest their functions and relevance to life activities.

A common concern is the difference between a CSF-contacting nucleus and a non-CSF-contacting nucleus. The DRN is a non-CSF-contacting nucleus and one of the most representative central nuclei. The differences in the location, structure, and morphology of these two nuclei have been clearly described in our previous papers [11, 23]. The most essential difference is that there are no CSF-contacting neurons in the DRN, and its processes do not extend into the CSF. In this study, a total of 913 differences were found by comparing the genes of the two nuclei. Of these, 607 were upregulated and 306 were downregulated in the CSF-contacting nucleus. These DEGs mainly indicate the binding sites between drugs and neuroactive substances, specific neural signaling pathways, and 5-HT synaptic transmission, among others. The results of this study further confirm that the CSF-contacting nucleus is unique at the genetic level.

We focused on the analysis of the top 40 highly-expressed genes in the CSF-contacting nucleus. The results suggest that these genes were mainly associated with energy metabolism, protein synthesis, assembly, operation, secretion, and hydrolysis. The most abundant neurotransmitter was serotonin (5-HT), and its gene expression was highly positively correlated with the number of neurons in the nucleus. 5-HT-ergic, GABAergic, cholinergic, and adrenergic receptors were also widely distributed. Cl^−^ [30, 31], Na^+^ [32–34], K^+^ [35, 36], Ca^2+^ [37, 38], and acid-sensing [39–41] channels have been routinely identified in the CSF-contacting nucleus. In particular, the ion channel genes of transient receptor potential (TRP) [42, 43] are highly expressed, suggesting that this nucleus may play an important role in nociception regulation [44, 45]. Several molecules with specific functional signaling pathways, such as CaMK, JAK, and MAPK, have been identified in the CSF-contacting nucleus. These molecules are involved in signaling pathways that often play an important role in neurosensitization and the synaptic transmission of information [46–48]. Based on the gene profile of the CSF-contacting nucleus, we also identified some genes associated with the regulation of specific physiology and diseases, such as brain development, immunity, Parkinson's disease, Alzheimer's disease, Huntington's disease, non-alcoholic fatty liver disease, choline metabolism in cancer, human papillomavirus infection, small RNA cancer, and small cell lung cancer. In particular, the solute carrier superfamilies are one of the most important membrane transport protein families on human cell membranes [49, 50] (including the inner membrane) expressed in the CSF-contacting nucleus. This strongly suggests that the CSF-contacting nucleus not only has a strong membrane-carrying capacity but also may be involved in the regulation of diabetes, hypertension, depression, and other diseases. These results provide new implications for the study of the mechanism and treatment of related diseases. Of course, these descriptions are just a few functional hints among the 19,666 genes of the CSF-contacting nucleus. Based on the gene profile of the CSF-contacting nucleus, we studied the relationship between several main genes, including the neurotransmitter 5-HT, GABA_B_ receptors, the peptide hormone ADM, and sodium ion channels in the CSF-contacting nucleus, and special life activities.

Antagonizing the 5-HT2_A_ receptor with ketanserin significantly increased the sensitivity to inflammatory pain, suggesting that 5-HT in the CSF-contacting nucleus is involved in pain regulation [18]. The expression of the GABA_B_ receptors in the CSF-contacting nucleus of rats was significantly decreased under chronic inflammatory pain induced by CFA. Activation of the GABA_B_ receptors in the CSF-contacting nucleus had a marked analgesic effect; in contrast, antagonizing the GABA_B_ receptors aggravated pain in rats, suggesting that the GABA_B_ receptor in the CSF-contacting nucleus plays an important role in the regulation of pain [20]. In addition, the expression of ADM, a molecule associated with chronic stress [51], was significantly increased in rats exposed to chronic restraint stress. After administration of the ADM receptor antagonist AM22-52, the stress behaviors of rats were aggravated. This result suggested that the upregulation of ADM in the CSF-contacting nucleus plays a compensatory role in the chronic stress response. Furthermore, the expression of Fos in the CSF-contacting nucleus increased significantly after the injection of hypertonic saline into the CSF. Both the spontaneous and induced sodium appetite of rats was enhanced after CSF-contacting nucleus KO. These results suggested that this nucleus can sense the change in sodium concentration in the CSF and plays an important role in sodium intake and desire for sodium [52].

In summary, these results strongly suggest that the CSF-contacting nucleus not only has the functional properties of general neurons but also has a strong capacity to sense, secrete, and transport and plays an important role in pain, stress, sodium desire, and many life activities.

### Limitations

Since 19,996 genes are involved in the CSF-contacting nucleus, the amount of information is enormous. In addition to genes, life activities and disease manifestations are also related to many factors, such as genomics, proteomics [53], and epigenetics [54]. Therefore, it is almost impossible to fully reveal the biological function of the whole nucleus in a limited paper. Here, we only describe the basic gene architecture of the nucleus and verify the relationship between the main genes and life activities. Much work remains to be done to fully understand the biological function of the CSF-contacting nucleus.
